# Identifying immune cell infiltration and effective diagnostic biomarkers for ischemic stroke using bioinformatics analysis

**DOI:** 10.1371/journal.pone.0310108

**Published:** 2024-12-05

**Authors:** Zongyong Zhang, Zongqing Zheng, Wenwei Luo, Jiebo Li, Jiushan Liao, Fuxiang Chen, Dengliang Wang, Yuanxiang Lin

**Affiliations:** 1 Department of Neurosurgery, The First Affiliated Hospital, Fujian Medical University, Fuzhou, China; 2 Department of Neurosurgery, National Regional Medical Center, Binhai Campus of the First Affiliated Hospital, Fujian Medical University, Fuzhou, China; 3 Fujian Provincial Institutes of Brain Disorders and Brain Sciences, First Affiliated Hospital, Fujian Medical University, Fuzhou, China; 4 Department of Neurosurgery, Nanping First Hospital Affiliated to Fujian Medical University, Fuzhou, China; 5 Department of Neurosurgery, Luoyuan County Hospital, Fuzhou, China; UT Health San Antonio: The University of Texas Health Science Center at San Antonio, UNITED STATES OF AMERICA

## Abstract

Ischemic stroke (IS) is a leading cause of death and disability worldwide. Screening for marker genes in IS is crucial for its early diagnosis and improvement in clinical outcomes. In the study, the gene expression profiles in the GSE22255 and GSE37587 datasets were extracted from the public database Gene Expression Omnibus. Weighted gene co‑expression network analysis (WGCNA) was used to investigate the gene sets that were related to ubiquitination. A total of 33 ubiquitination-related differentially expressed genes (DEGs) were identified using “limma (version 3.50.0)”. Gene set enrichment analysis (GSEA) and gene set variation analysis (GSVA) analysis enriched multiple pathways that were closely related to IS. The correlations between the HALLMARK signaling pathways and DGEs were analyzed. Receiver operating characteristic analysis was used to validate the diagnostic value of the key genes. Among them, 16 genes were identified as hub genes. Single-sample GSEA was performed to evaluate the infiltration status of immune cells in IS. To understand the potential molecular mechanisms of the hub genes in IS, we constructed RBP-mRNA and mRNA–miRNA–lncRNA interaction networks. Additionally, we used the GeneMANIA database to create a PPI network for the signature genes to investigate their functions. As a result, there was a significant difference in the overall infiltration of immune cells between the IS and control groups. Among the 28 types of immune cells, the degree of infiltration of seven types was significantly different between the two groups (*p*<0.05). The expression of four types of immune cells, namely type 1 T helper cell, type 17 T helper cell, eosinophil, and mast cell, in the IS group were significantly higher than that in the control group. The expressions of DHFR2 (R = -0.575; *p*<0.001) and DNAAF2 (R = -0.562; *p*<0.001) were significantly negatively correlated with eosinophil infiltration. The PPI network demonstrated that the 16 hub genes interacted with each other. In conclusion, we identified DEGs, WGCNA modules, hub genes, enriched pathways, and infiltrating immune cells that may be closely involved in IS. Further studies are required to explore the functions of these genes.

## 1 Introduction

As the second leading cause of death and disability worldwide, stroke causes 5.5 million deaths and 102 million disabilities annually [1, 2]. Ischemic stroke (IS) accounts for approximately 80% of all occurrences of stroke [3]. Currently, the only therapeutic method to reduce the IS-related brain damage and improve its clinical outcomes is recanalization of the occluded vessels by pharmacological treatment using tissue plasminogen activator or thrombectomy [4]. In terms of reperfusion injury, the earlier blood flow recovery the better the clinical outcomes [5]. However, head CT may not detect infarcted lesions in the early stages (within 6 h), whereas MRI is difficult to obtain in a timely manner for various reasons; this in turn which may lead to misdiagnosis or delayed diagnosis with a subsequent delay in receiving the optimum treatment intervention. Consequently, the early diagnosis and treatment of IS are challenging [6]. Therefore, screening for marker genes is crucial for its early diagnosis, discovery of new therapeutic targets, and improvement of clinical treatment outcomes.

Ubiquitination is an efficient and precise post-translational modification that affects the stability, localization, dynamic interactions, and corresponding functions of target proteins labeled with ubiquitin. Ubiquitination was previously believed to only act as a signal for protein degradation. However, it has recently been shown to be crucial for various cellular processes, including inflammation, apoptosis, cell cycle regulation, enzyme activation, signal transduction, transcription, and DNA repair [7]. Ubiquitination is directly induced by reperfusion after ischemia and can be rapidly detected in neurons around the infarcted area [8]. Given the importance of ubiquitination in cellular homeostasis and stress response, this change may significantly affect neuronal survival and function during IS. Therefore, we need to further explore the mechanisms underlying the ubiquitination of the proteins involved in IS.

Physiologically, the infiltration of peripheral immune cells into the central nervous system is controlled by the blood-brain barrier and regulated by endothelial-immune interactions [9]. Following ischemic stroke, energy consumption and hypoxia lead to neuronal death; thus, activating resident glial cells and promoting the infiltration of peripheral immune cells into the brain with various subsequent immune-mediated and even contradictory effects [9]. The immune response is closely involved in all the ischemic cascade stages, starting from acute ischemic brain parenchymal damage to subsequent tissue repair [10]. Immune cell infiltration induces neuronal apoptosis; exacerbates ischemic damage; and promotes neuronal repair, differentiation, and regeneration [11]. Few studies have investigated the infiltration patterns of immune cells in IS. Therefore, evaluating the infiltration patterns of immune cells in the peripheral blood of patients with IS will help elucidate the immune-related molecular mechanisms in IS.

Recently, bioinformatics methods have been widely used to analyze high-throughput and microarray data to identify the differentially expressed genes (DEGs) and perform various analyses. Additionally, these methods have shown superior ability with respect to identifying the potential mechanisms underlying various human diseases. Thus, this study aimed to explore putative significant genes, critical modules, pathways, and infiltrating immune cells implicated in the pathogenesis of IS, based on a comprehensive genomic analysis of the publicly available datasets.

## 2 Materials and methods

### 2.1 Data sources and preprocessing

All the data used in this study are freely accessible to the public, mainly derived from the Gene Expression Omnibus (GEO, https://www.ncbi.nlm.nih. gov/geo/). The whole genome-wide expression profiles of IS was retrospectively downloaded using the R package “GEOquery” from the GEO (https://www.ncbi.nlm.nih.gov/geo/) database. The GSE22255 database was sequenced based on GPL570 [HG-U133_Plus_2] Affymetrix Human Genome U133 Plus 2.0 Array, included 20 patients with IS and 20 controls. The GSE37587 dataset was sequenced based on GPL6883 Illumina HumanRef-8 v3.0 expression beadchip, included 68 patients with IS (Table 1). Batch effects from non-biological technical biases were corrected using the ComBat method of the R package “sva” [12]. Principal component analysis (PCA) was performed to examine the degree of correction. This study honors the data access policies of each database. No ethics approval and patients’ informed consent were needed for this study.

**Table 1:** Data used in this study.

ID	Experimental Group	Control Group
GSE22255	20 patients with IS	20 controls
GSE37587	68 patients with IS.	N/A

Note: The above two datasets were combined into a single dataset containing 88 patients with IS and 20 controls after removing the batch effect and were used for all subsequent analyses.

### 2.2 DEGs associated with IS

Identification of DEGs contributes in identifying the main genes playing a driving role between the groups, the DEGs between the control (n = 20) and IS (n = 88) samples were identified using the “limma (version 3.50.0)” package in R with the thresholds of |log2Fold Change|>0.25 and an adjusted p-value<0.05 as previously reported [13]. Subsequently, the heatmap was generated using the R package “pheatmap” with Euclidean distance and complete linkage clustering method.

### 2.3 Gene set enrichment analysis (GSEA)

GSEA is a computational method that determines whether an a *priori* defined set of genes shows statistically significant concordant differences between two biological states [14]. GSEA was carried out using the R package “clusterProfiler (version 4.2.2)” on an ordered list of all genes based on their log2Fold Change values. Gene set permutations were performed 1,000 times in each analysis. We selected c2.cp.kegg.v7.5.1. symbols in the Molecular Signatures Database (MSigDB) Collections as the reference gene collection method [14–16]. A gene set with an adjusted p-value<0.05 was considered as significantly enriched.

### 2.4 Gene set variation analysis (GSVA)

GSVA is an unsupervised and non-parametric gene set enrichment method that permits the use of gene expression profiles to assess the associations between biological pathways and gene features. To investigate the difference in the biological function between the control and IS groups, GSVA was performed with “c2.cp.kegg.v7.5.1. symbols” using the R package “GSVA (version 1.42.0)”. The R package “pheatmap (version 1.0.12)” was used to visualize the results. A total of 50 hallmark gene sets were downloaded from the MSigDB database (http://software.broadinstitute.org/gsea/msigdb) as reference gene sets. We used the single-sample GSEA (ssGSEA) function in the GSVA package to calculate the GSVA score for each gene set in the different samples. The “Limma” package was used to compare the differences in the GSVA scores of the different gene sets between the control and IS groups [13].

### 2.5 Weighted gene co‑expression network analysis (WGCNA) and identification of significant modules

WGCNA is a systems biology method used to describe the synergistically change in gene sets and the relationship between these change patterns and traits. This helps identify gene modules that play a key role in biological processes and diseases by constructing gene co-expression networks to reveal how genes work in harmony. Co-expression networks were constructed using the WGCNA algorithm implemented in the R “WGCNA” package (version 1.70–3) [17]. The Pearson correlation coefficient was calculated to assess the similarity in the gene expression profiles, and the correlation coefficients between the genes were weighted using a power function to obtain a scale-free network. Using the R package ‘PickSoftThreshold’, we established a weighted adjacency matrix by increasing the co-expression similarity to a power β of 8. A gene module is a cluster of co-expressed densely interconnected genes. WGCNA uses hierarchical clustering to identify gene modules and colors to indicate the modules. The dynamic tree cut method was used to identify different modules, during which the adjacency matrix, a measure of topology similarity, was converted to a topology overlay matrix and modules were detected using cluster analysis. The significant associations of module eigengene (the first principal component of the module and represents its overall expression level) with ubiquitination were calculated using Pearson’s correlation analysis. The structure of the co-expression module was visualized using heat map plots of topological overlap in the gene network. The relationships among the modules were summarized using a hierarchical clustering dendrogram of the eigengenes and heatmap plot of the corresponding eigengene network. The ubiquitination-related DEGs were obtained from the intersection of DEGs and genes from the ubiquitination-related module.

### 2.6 Gene Ontology (GO) and Kyoto Encyclopedia of Genes and Genomes (KEGG) pathway enrichment analyses

GO and KEGG enrichment assays are two methods commonly used to understand the distribution of gene sets in biological processes, molecular functions, or cellular components, as well as their involvement in specific metabolic pathways or signaling pathways. GO enrichment analysis includes biological process (BP), molecular function (MF), and cellular component (CC) analyses [18]. The R package “clusterProfiler” (version 4.2.2) was used to perform GO enrichment analysis (p-value<0.05) on the ubiquitination-related DEGs [19].

### 2.7 GeneMANIA

GeneMANIA is an online tool for gene function prediction and gene network analysis. It analyzes multiple association types between genes to predict gene function and construct gene networks, helping researchers identify possible functional associations and biological pathways in gene lists. The protein–protein interaction (PPI) networks of the hub genes were constructed using the GeneMANIA website (http://genemania.org) which can also predict the relationships between functionally similar and hub genes [20], including protein-protein and protein-DNA interactions, pathways, physiological and biochemical reactions, co-expression, and co-localization.

### 2.8 The receiver operating characteristic (ROC) curve

The ROC curve, which is defined as a plot of test sensitivity as the y-coordinate versus its 1-specificity or false positive rate as the x-coordinate, is an effective method for evaluating the performance of diagnostic tests. The area under the curve (AUC) is the most common metric obtained from the ROC plot of sensitivity against 1-specificity. We used the R package “pROC” to create ROC curves, determine the AUC for screening signature genes, and evaluate their diagnostic values [21]. Thus, it is measured on a scale of 0.5 (coin flip) to 1 (perfect differentiation). In general, an AUC value of 0.5 indicates no differentiation, 0.6–0.8 indicates acceptable, 0.8–0.9 indicates excellent, and more than 0.9 indicates outstanding.

### 2.9 Immune infiltration analysis

Immune infiltration evaluates the distribution and accumulation of immune cells in tumor tissues by analyzing RNA sequencing data to assess the role of the immune system in the occurrence of inflammation-related diseases. ssGSEA [22], an extension of GSEA, calculates separate enrichment scores for each sample-gene set pairing. Each ssGSEA enrichment score represents the degree to which the expression levels of genes in a particular gene set are coordinately up- or down-regulated within a sample. ssGSEA is a variation of the GSEA algorithm; it provides a score for each sample and gene set pair instead of calculating the enrichment scores for groups of samples (i.e., control vs. disease) and sets of genes (i.e., pathways).

The relative enrichment score of each respective immunocyte was quantified from the gene expression profile of each sample based on the 28 types of immune cells, namely activated CD8 T cell, central memory CD8 T cell, effector memory CD8 T cell, activated CD4 T cell, central memory CD4 T cell, effector memory CD4 T cell, T follicular helper cell, gamma delta T cell, type 1 T helper cell, type 17 T helper cell, type 2 T helper cell, regulatory T cell, activated B cell, immature B cell, memory B cell, natural killer cell, CD56bright natural killer cell, CD56dim natural killer cell, myeloid derived suppressor cell, natural killer T cell, activated dendritic cell, plasmacytoid dendritic cell, immature dendritic cell, macrophage, eosinophil, mast cell, monocyte and neutrophil, downloaded from the TISIDB (Tumor and Immune System Interactions Database) (http://cis.hku.hk/TISIDB/index.php) [23]. Variations in immune cell infiltration levels among samples from the IS and control groups were illustrated using the R package ggplot2 (version 3.3.6) [24].

### 2.10 Statistical analyses

Statistical analyses were performed using the R software v4.1.2. Spearman’s correlation test was used to infer correlations between two parameters. Wilcoxon test was adopted to compare differences between two groups, while Kruskal–Wallis tests was performed to compare differences among three or more groups. Statistical significance was set at a *p*<0.05.

## 3 Results

### 3.1 WGCNA and module identification

**Fig 1:**
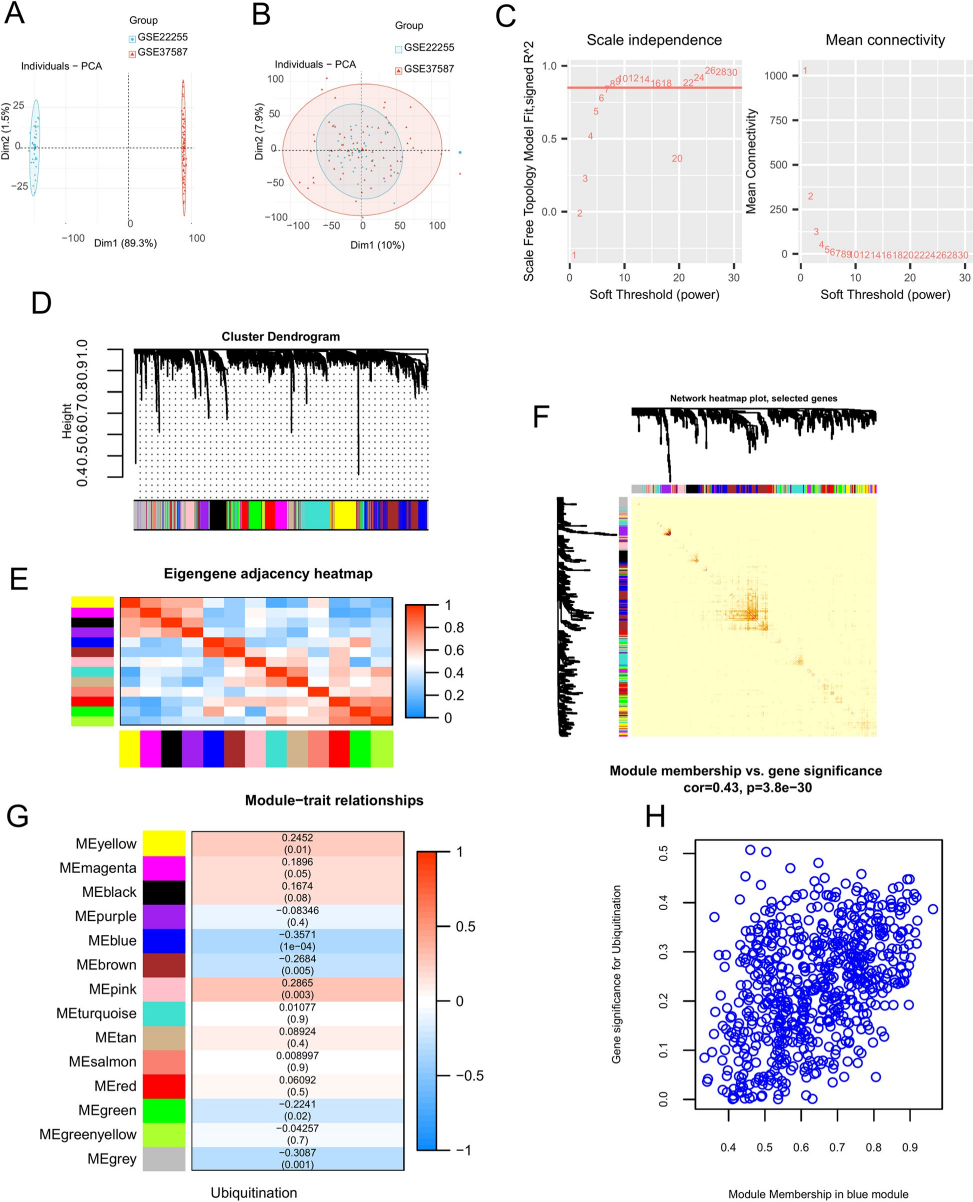
Construction of WGCNA co-expression network. (A) Data before removing batch effects. (B) Data after removing batch effects. (C) Soft threshold β = 8 and scale-free topological fit index (R2). (D) Network analysis of gene expression in IS identifies distinct modules of co-expression data. (E) Relationships among modules. Heatmap plot of the adjacencies in the eigengene network. Each row and column in the heatmap corresponds to one module eigengene (labeled by color). In the heatmap, the red color represents high adjacency, while the blue color represents low adjacency. Squares of red color along the diagonal represent the meta-modules. (F) Heatmap plot of topological overlap in the gene network. In the heatmap, each row and column corresponds to a gene, the light color denotes low topological overlap, and the progressively darker red color denotes higher topological overlap. Dark squares along the diagonal correspond to modules. The gene dendrogram and module assignment are shown along the left and top. (G) Relationships between consensus module eigengenes and ubiquitination. Each row in the table corresponds to a consensus module, and each column corresponds to a sample or trait. Numbers in the table report the correlations between the corresponding module eigengenes and traits, with the p-values presented below the correlations in parentheses. The table is color coded based on correlation according to the color legend. (H) Correlation between module membership (MM) and gene significance (GS) for ubiquitination of all genes in the blue module. ‘Cor’ represents absolute correlation coefficient between GS and MM.

We combined data from GSE22255 and GSE37587 for subsequent analysis. The PCA results showed that the datasets were independent of each other and exhibited strong batch effects (Fig 1A), these differences were subsequently removed (Fig 1B). The two datasets were merged into a new dataset consisting of 88 patients with IS and 20 controls, which was used for all subsequent analyses. WGCNA was used to investigate the gene sets that were related to ubiquitination. The scale independence and mean connectivity analyses showed that when the weighted value was equal to 8 (Fig 1C), the average degree of connectivity was close to 0 and the scale independence was>0.85. Thirteen co-expressed modules were identified, and uncorrelated genes were assigned to a gray module, which was ignored in the subsequent analysis (Fig 1D). To study the relationships between the modules and determine their correlations, we correlated the MEs. A heatmap of the eigengene network is shown in Fig 1E. A heatmap of the topological overlap in the gene network is shown in Fig 1F. To understand the physiological significance of these modules, we correlated 13 MEs with ubiquitination and searched for the most significant associations. According to the heatmap of the module-trait correlation (Fig 1G), genes clustered in the blue module (n = 639) had the strongest negative correlation with ubiquitination (r = -0.3571; *p*<0.05). Thus, the blue module was mainly considered in the subsequent analysis because it may accurately indicate ubiquitination. Fig 1H shows the scatterplots of gene significance (GS) for ubiquitination versus the module membership (MM), which is present in the blue module. MM and GS for ubiquitination exhibited significant positive correlations (cor = 0.43, *p*<0.05), implying that the most important (central) elements of the blue module tended to be highly correlated with ubiquitination.

### 3.2 Identification of DEGs

**Fig 2:**
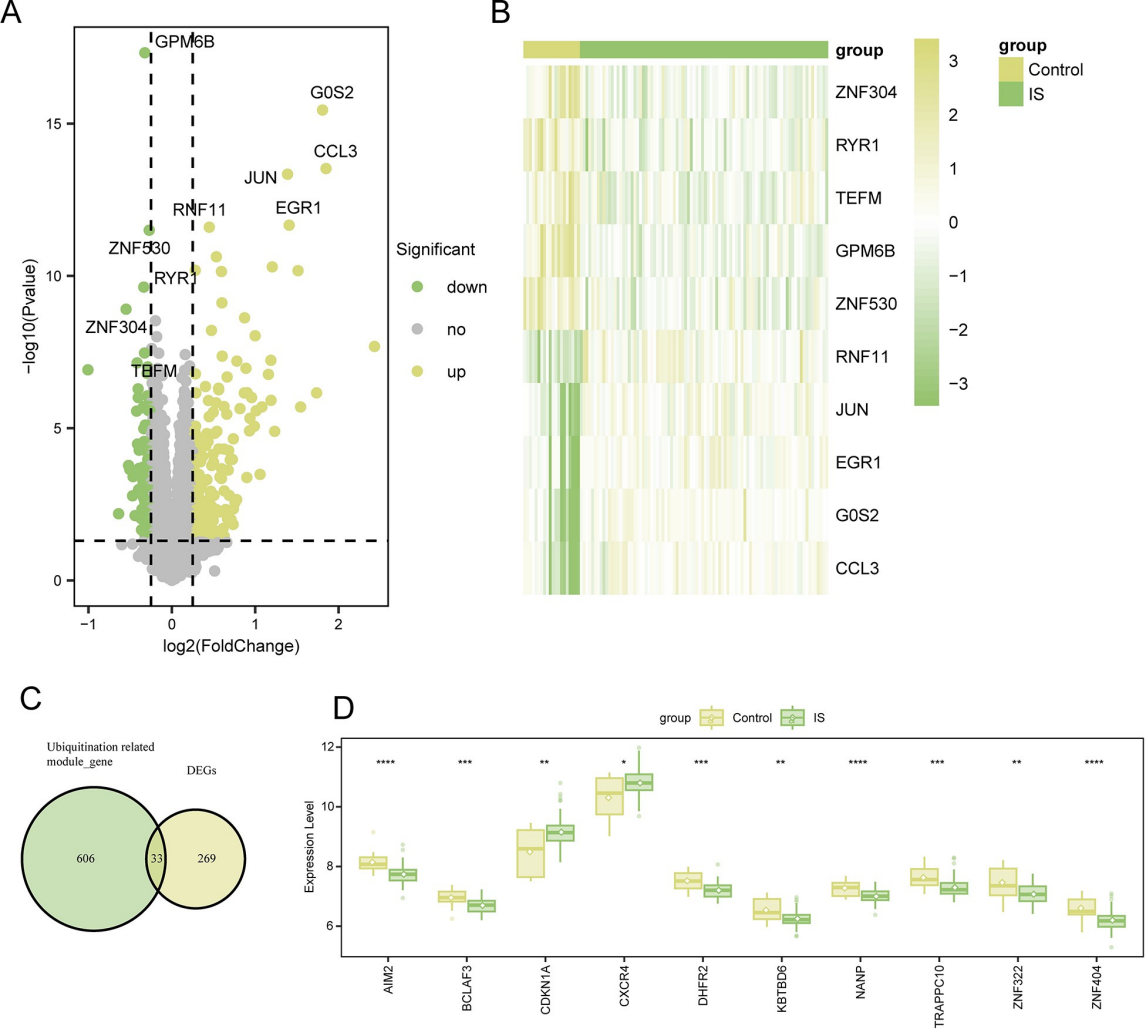
DEGs identification. (A) A volcano plot depicting the distribution of DEGs between the IS and control samples. Yellow, green, and gray dots represent gene expression levels corresponding to the upregulated, downregulated, and insignificant expression, respectively. (B) A heatmap depicting the top 5 DEGs with upregulated expression and top 5 DEGs with downregulated expression. (C) Venn plot shows the interaction between the DEGs and module genes. (D) The variations in the expression levels of the top 10-gene between IS and control groups were determined using the Wilcoxon tests. The asterisks represented the p-values (****p<0.0001, ***p<0.001, **p<0.01, *p<0.05).

Based on the comparison of the IS samples and controls, 302 DEGs were identified as statistically significant between the two groups (adjusted *p*<0.05, |Log2-fold change|>0.25). In the IS samples, expression of 200 and 102 genes was upregulated and downregulated, respectively. All DEGs were visualized using a volcano plot (Fig 2A). Furthermore, the top 5 genes with upregulated expression (R*NF11*, *JUN*, *EGR1*, *G0S2*, and *CCL3*) along with the top 5 DEGs with downregulated expression (*ZNF304*, *RYR1*, *TEFM*, *GPM6B*, and *ZNF530*) were shown in the heatmap in Fig 2B.

A total of 33 ubiquitination-related DEGs were obtained from the intersection of DEGs and ubiquitination-related module genes, which were considered to be key genes (Fig 2C). Wilcoxon test revealed the top 10 genes that showed significant differences in their expression levels between the two groups (*p*<0.05; Fig 2D).

### 3.3 GSEA

**Fig 3:**
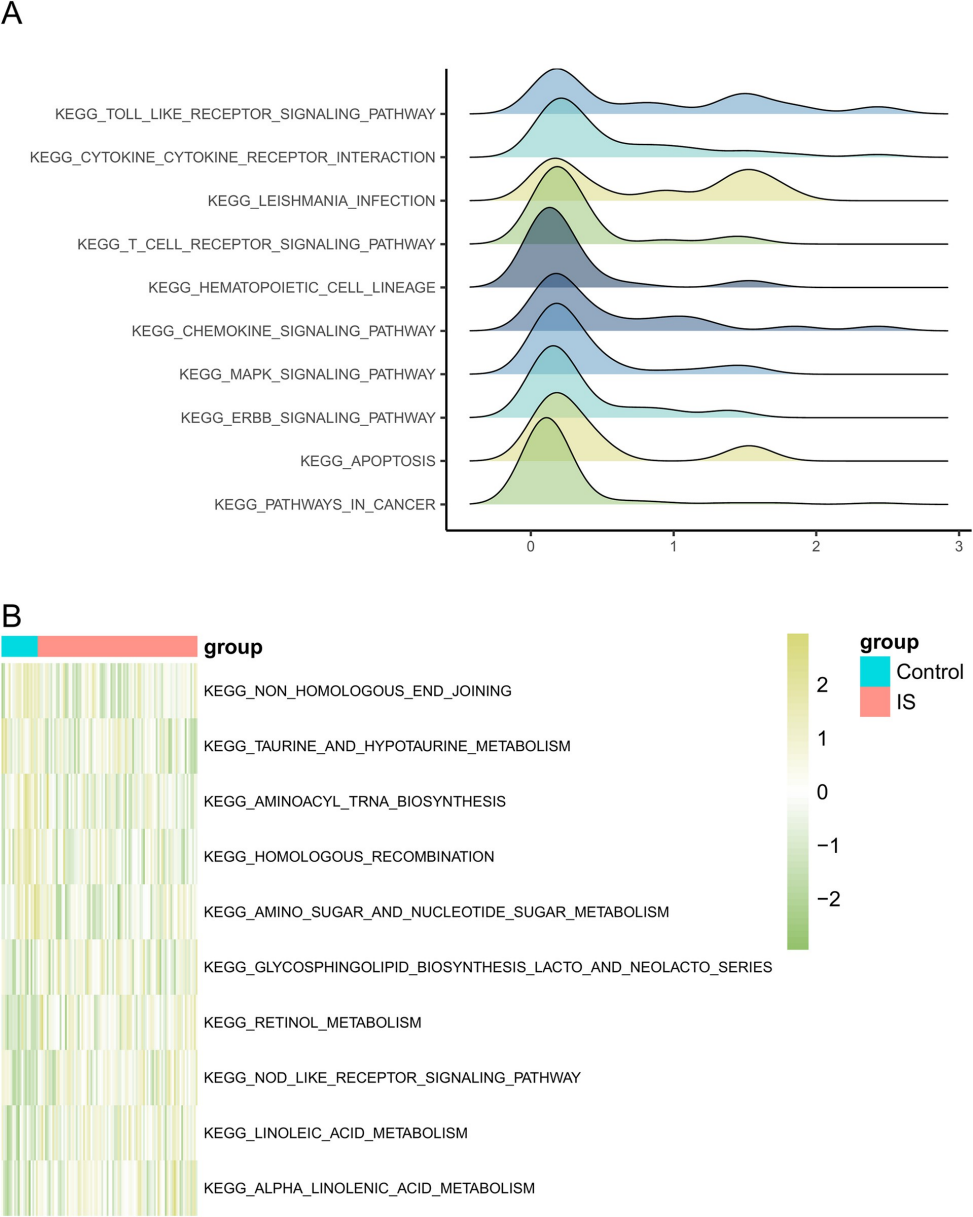
Significantly enriched pathways. (A) GSEA ridge plot. (B) The heatmap illustrates the GSVA analysis results.

GSEA was performed to further explore the potential mechanisms underlying the DEGs. Using the MSigDB Collection, the most significantly enriched signaling pathways were selected based on their normalized enrichment scores (NES). GSEA identified the following significantly enriched signaling pathways in IS KEGG_PATHWAYS_IN_CANCER (NES = 1.689; adjusted *p* = 0.025; false discovery rate [FDR] = 0.02), KEGG_MAPK_SIGNALING_PATHWAY (NES = 1.906; adjusted p = 0.025; FDR = 0.02), KEGG_CYTOKINE_CYTOKINE_RECEPTOR_INTERACTION (NES = 2.238; adjusted *p* = 0.025; FDR = 0.02), KEGG_CHEMOKINE_SIGNALING_PATHWAY (NES = 2.016; adjusted P = 0.025; FDR = 0.02), KEGG_T_CELL_RECEPTOR_SIGNALING_PATHWAY (NES = 2.1; adjusted *p* = 0.025; FDR = 0.02), KEGG_APOPTOSIS (NES = 1.72; adjusted p = 0.025; FDR = 0.02), KEGG_TOLL_LIKE_RECEPTOR_SIGNALING_PATHWAY (NES = 2.384; adjusted p = 0.025; FDR = 0.02), KEGG_ERBB_SIGNALING_PATHWAY (NES = 1.802; adjusted *p* = 0.025; FDR = 0.02), KEGG_HEMATOPOIETIC_CELL_LINEAGE (NES = 2.082; adjusted *p* = 0.025; FDR = 0.02), and KEGG_LEISHMANIA_INFECTION (NES = 2.22; adjusted *p* = 0.025; FDR = 0.02) (Fig 3A).

### 3.4 GSVA

To further explore the functional annotation between the IS and control samples, GSVA was performed to evaluate the difference in the relative expression between the pathways in the two groups. GSVA enriched many differentially expressed pathways that were visualized using a heat map. The expression of the pathways KEGG_TAURINE_AND_HYPOTAURINE_METABOLISM and KEGG_AMINOACYL_TRNA_BIOSYNTHESIS was significantly lower in the IS group than in the control group, whereas the expression of KEGG_GLYCOSPHINGOLIPID_BIOSYNTHESIS_LACTO_AND_NEOLACTO_SERIES and KEGG_ALPHA_LINOLENIC_ACID_METABOLISM-associated pathways was significantly higher in the IS group than in the control group (Fig 3B).

### 3.5 Validation of the hub genes

**Fig 4:**
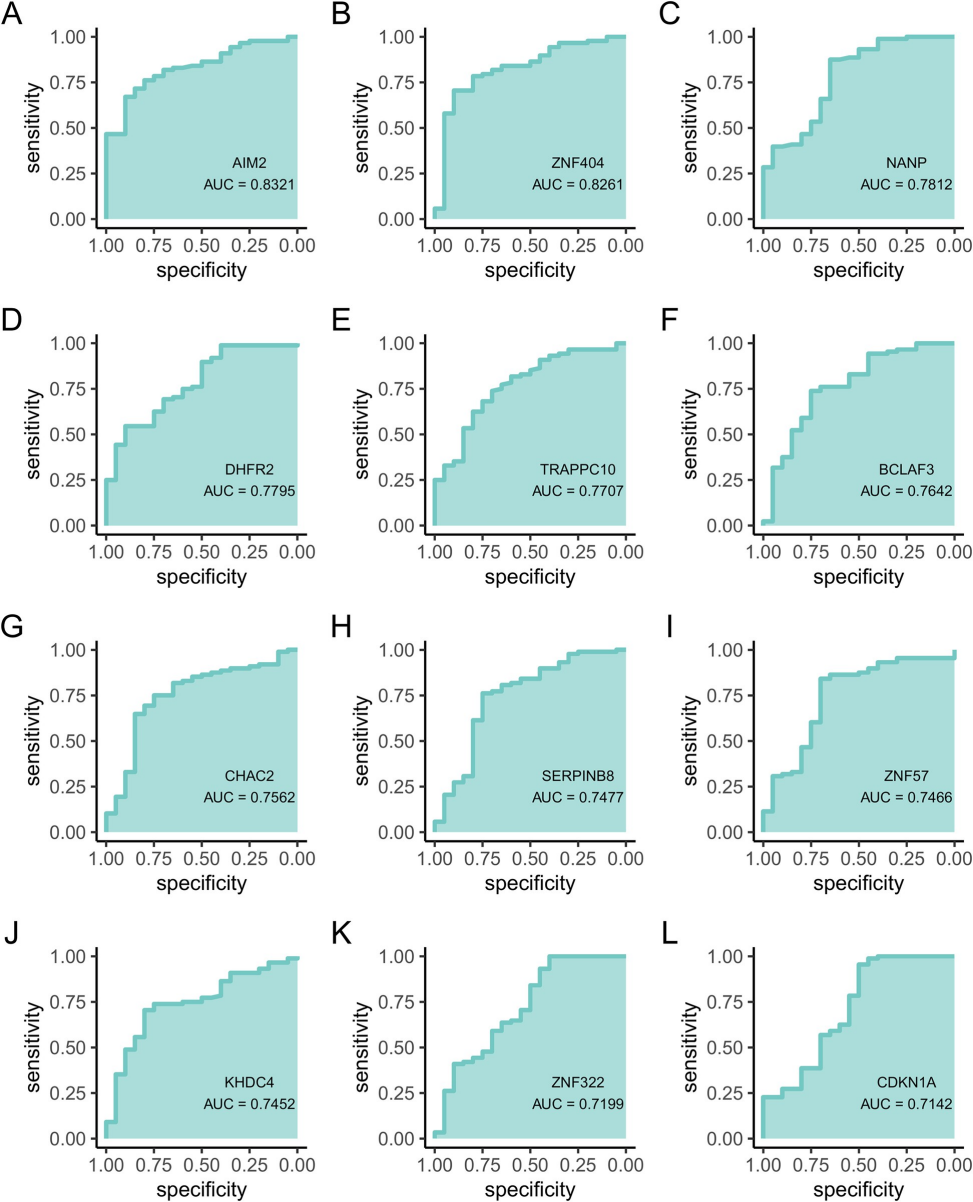
ROC curves of the hub genes. ROC curves of (A) AIM2. (B) ZNF404. (C) NANP. (D) DHFR2. (E) TRAPPC10. (F) BCLAF3. (G) CHAC2. (H) SERPINB8. (I) ZNF57. (J) KHDC4. (K) ZNF322. (L) CDKN1A.

ROC analysis was used to validate the diagnostic value of the key genes. The hub genes AIM2(AUC = 0.832), ZNF404(AUC = 0.826), NANP(AUC = 0.781), DHFR2(AUC = 0.78), TRAPPC10(AUC = 0.771), BCLAF3(AUC = 0.764), CHAC2(AUC = 0.756), SERPINB8(AUC = 0.748), ZNF57(AUC = 0.747), KHDC4(AUC = 0.745), ZNF322(AUC = 0.72), CDKN1A(AUC = 0.714), TRMT1L(AUC = 0.713), KBTBD6(AUC = 0.708), DNAAF2(AUC = 0.707), and PRKAR2B(AUC = 0.702) had similar AUC values (Fig 4A–4L and S1 Fig), indicating that the identified hub genes demonstrated an acceptable differentiation capability as potential biomarkers for IS.

### 3.6 Immune cells infiltration

**Fig 5:**
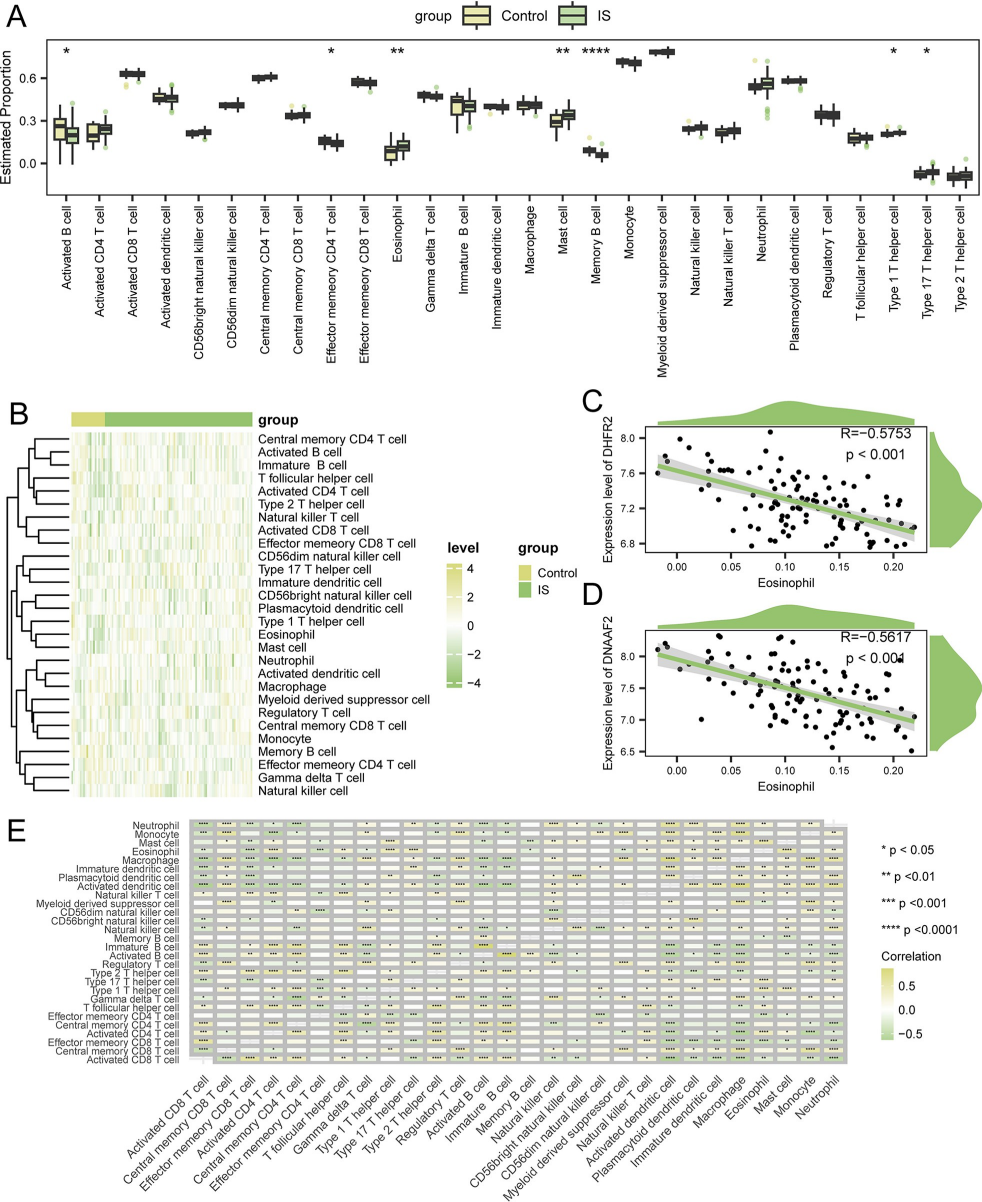
Difference in immune infiltrations between the IS and control samples. (A) The estimated proportions of infiltrating immune cells in the IS and control groups. (B) The heatmap presenting changes in immune infiltration levels between the IS and control groups. (C) Correlations between DHFR2 and eosinophils. (D) Correlation between DNAAF2 and eosinophils. (E) Correlation among the immune cells. The asterisks represent the p-values (****p<0.0001, ***p<0.001, **p<0.01, *p<0.05).

Immune cell infiltration plays an essential role in the pathogenesis of IS. Therefore, we investigated the association between IS and control samples and infiltrating immune cells. Among the 28 types of immune cells, the degree of infiltration of 7 types was significantly different between the two groups (*p*<0.05; Fig 5A). The degree of infiltration of 4 types of immune cells (type 1 T helper cells, type 17 T helper cells, eosinophils, and mast cells) was significantly higher in the IS group than in the control group. The overall infiltration levels of immune cells varied markedly between the IS and control groups (Fig 5B).

Furthermore, significant correlations between each hub gene and the corresponding immune cells were detected. DHFR2 and DNAAF2 were significantly associated with eosinophils (r = -0.575, *p*<0.001; Fig 5C and r = -0.562, *p*<0.001; Fig 5D) respectively. Subsequently, the correlations between each infiltrated immune cell type were estimated. Most immune cells were positively correlated with each other (Fig 5E).

### 3.7 Signaling pathways involved in signature genes

**Fig 6:**
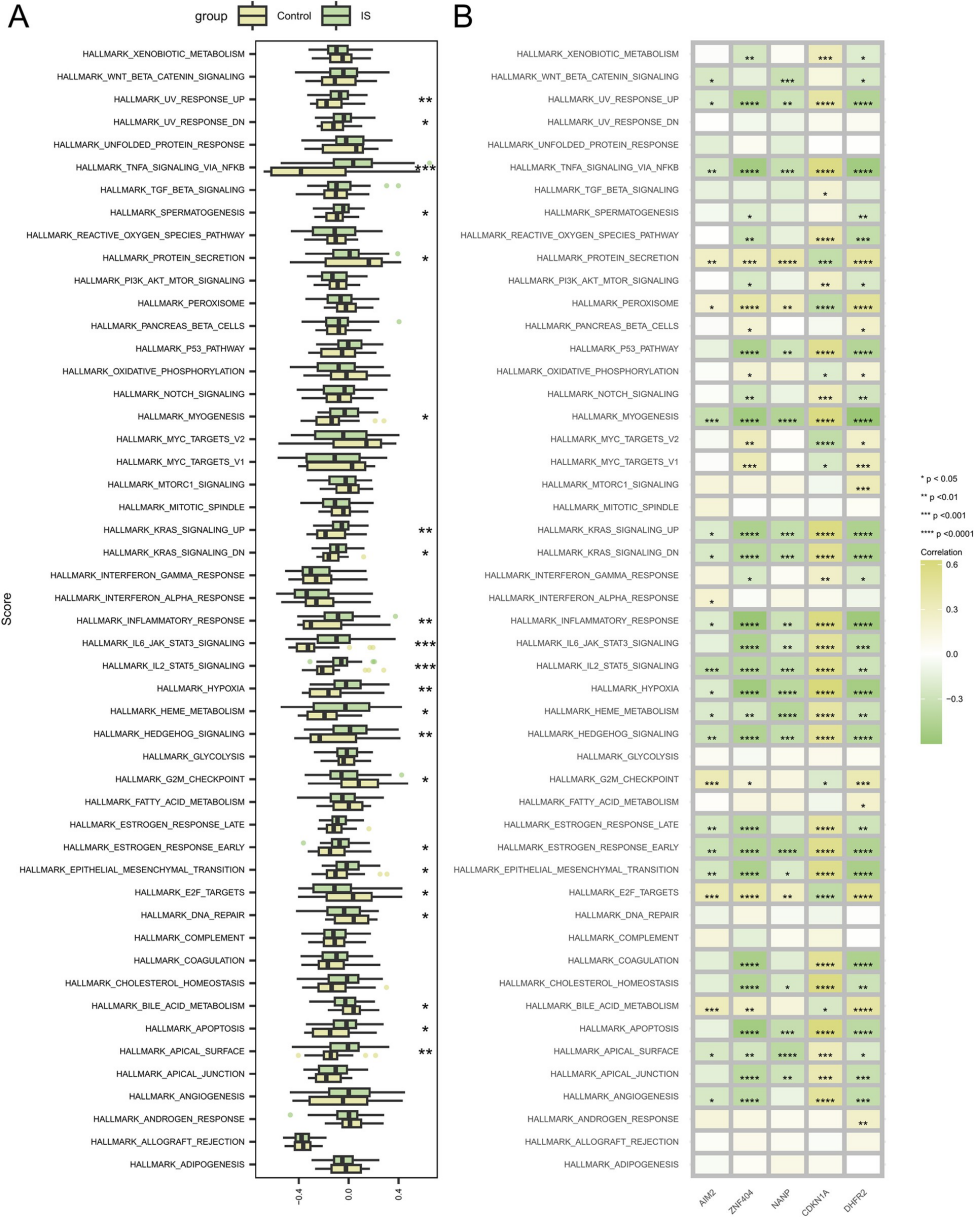
Correlation between the hub genes and 50 hallmark signaling pathways. (A) Comparison of the 50 HALLMARK signaling pathways between the IS group and controls. (B) Correlation between the hub genes and the 50 hallmark signaling pathways. The asterisks represent the p-values (****p<0.0001, ***p<0.001, **p<0.01, *p<0.05).

The differences in the 50 hallmark signaling pathways between patients with IS and controls were further investigated using GSVA. In the patients with IS, the levels of 17 hallmark signaling pathways, namely HALLMARK_APICAL_SURFACE, HALLMARK_APOPTOSIS, HALLMARK_EPITHELIAL_MESENCHYMAL_TRANSITION, HALLMARK_ESTROGEN_RESPONSE_EARLY, HALLMARK_HEDGEHOG_SIGNALING, HALLMARK_HEME_METABOLISM, HALLMARK_HYPOXIA, HALLMARK_IL2_STAT5_SIGNALING, HALLMARK_IL6_JAK_STAT3_SIGNALING, HALLMARK_INFLAMMATORY_RESPONSE, HALLMARK_KRAS_SIGNALING_DN, HALLMARK_KRAS_SIGNALING_UP, HALLMARK_MYOGENESIS, HALLMARK_SPERMATOGENESIS, HALLMARK_TNFA_SIGNALING_VIA_NFKB, HALLMARK_UV_RESPONSE_DN, and HALLMARK_UV_RESPONSE_UP, were significantly up-regulated. The levels of five pathways, namely HALLMARK_BILE_ACID_METABOLISM, HALLMARK_DNA_REPAIR, HALLMARK_E2F_TARGETS, HALLMARK_G2M_CHECKPOINT, and HALLMARK_PROTEIN_SECRETION, were significantly down-regulated in the patients with IS (Fig 6A).

The correlations of the 5 most-significant differentially expressed hub genes were analyzed using 50 HALLMARK signaling pathways (Fig 6B). AIM2 is associated with several pathways, including those involving type 1 T helper cells and memory CD4 T cells. ZNF404 is also associated with many other pathways, including those involving eosinophils and mast cells. This further confirmed that these genes may be involved in the regulation of the related pathways in IS.

### 3.8 Construction and functional annotation of the crosstalk between the hub mRNAs and RNA binding proteins (RBPs)

**Fig 7:**
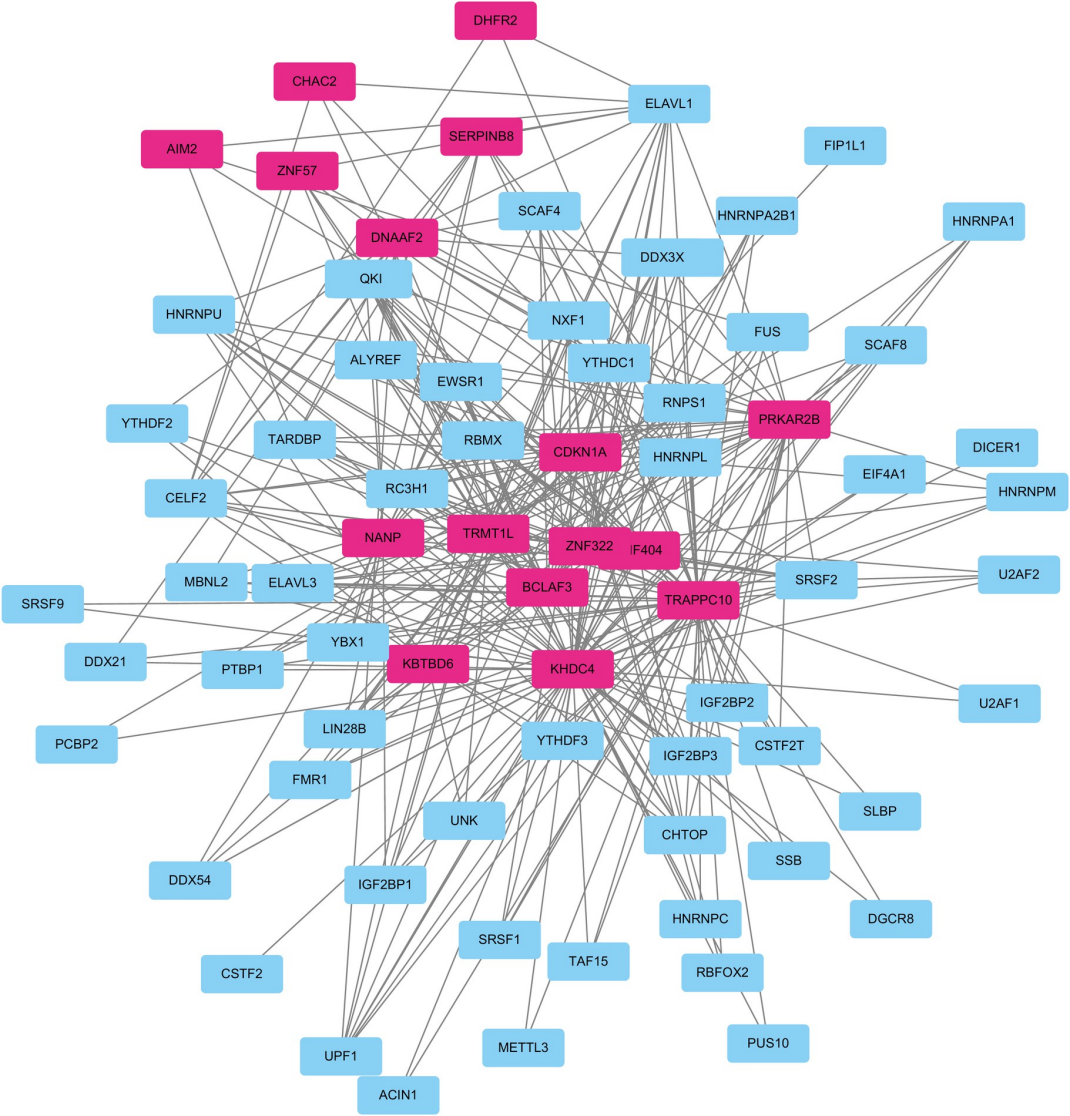
The RBP–mRNA regulatory network; the blue and pink colors represent the RBPs and mRNAs, respectively.

Based on the fact that RBPs bind to mRNA, we searched for 16 hub mRNAs using StarBase. The search yielded and 16 mRNA-RBP pairs, which were then downloaded. Based on the relationships between the target genes provided by the online dataset, we constructed an RBP-mRNA network comprising 74 nodes, 58 RBPs, 16 mRNAs, and 325 edges. The PPI network is shown in Fig 7.

### 3.9 Construction of the competitive endogenous RNA network

**Fig 8:**
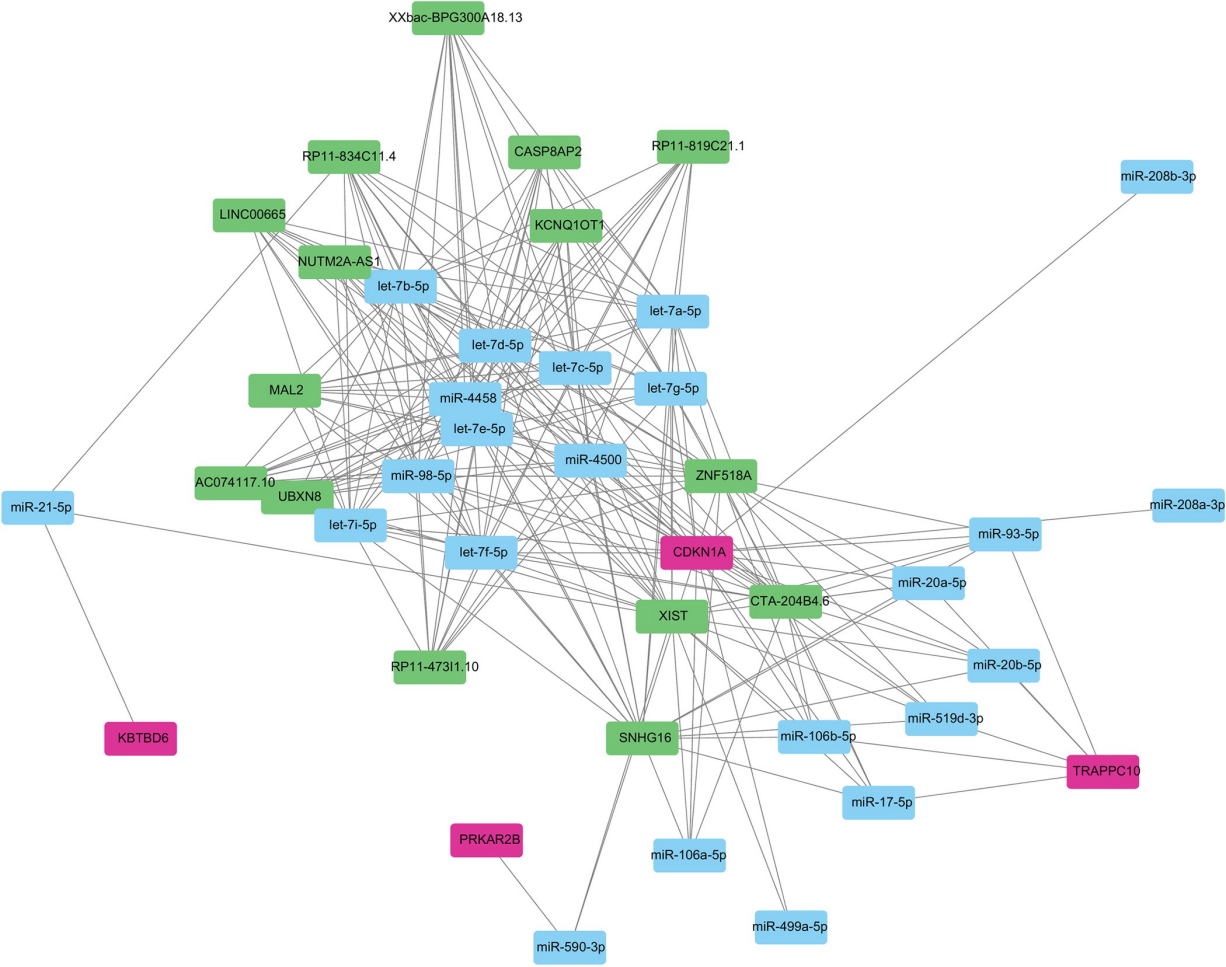
Construction of lncRNA-miRNA-mRNA network; the green, pink, and blue rectangles represent the lncRNA, mRNA, and miRNA, respectively.

To understand the potential molecular mechanism of the hub genes in IS, we constructed an mRNA–miRNA–lncRNA interaction network. In total, 4 mRNA notes, 15 lncRNA notes, 23 miRNA notes, and 227 edges were constructed in the mRNA–miRNA–lncRNA interaction network (Fig 8).

### 3.10 Trait gene interaction analysis

**Fig 9:**
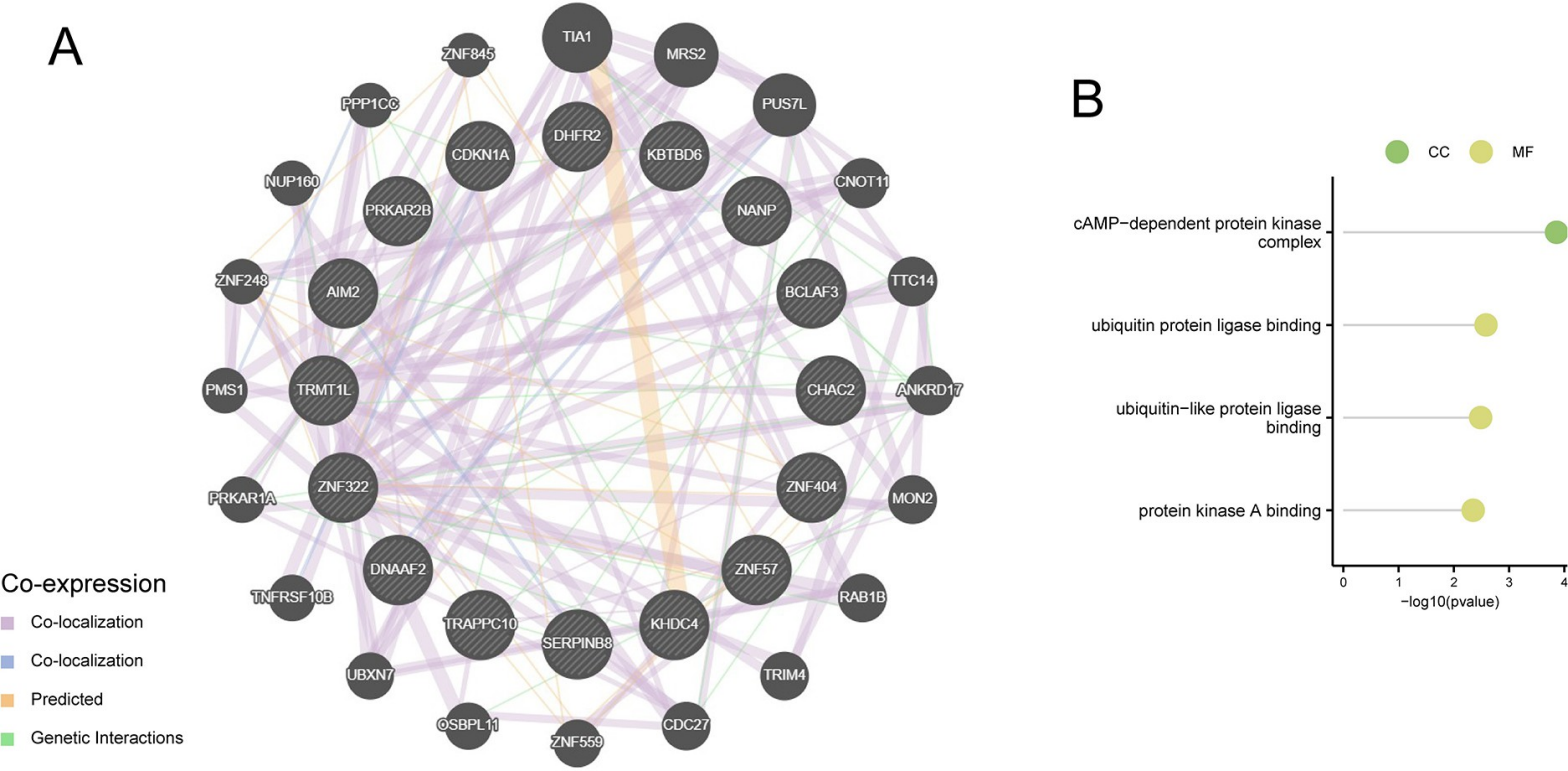
Interaction analysis of the hub genes. (A) Characterized gene co-expression network. (B) GO analysis of the co-expressed genes.

We used the GeneMANIA database to create a PPI network for the signature genes and identified 16 genes in the PPI network (Fig 9A). To further investigate the functions of the signature genes, GO and KEGG analyses were performed on 36 genes, including 16 hub and 20 related genes. GO results revealed that these genes were strongly enriched in the cAMP-dependent protein kinase complex (GO:0005952; CC), ubiquitin protein ligase binding (GO:0031625), ubiquitin-like protein ligase binding (GO:0044389), and protein kinase A binding (GO:0051018; MF; Fig 9B).

## 4 Discussion

Due to the globally aging population, an increasing number of people suffer from IS, which poses huge social and economic burdens [2]. Currently, early drug thrombolysis and mechanical thrombectomy are the only effective measures for improving the clinical prognosis. However, owing to the lack of early diagnostic indicators, patients with IS often do not receive the best treatment, leading to poor prognosis. In addition, immune cell infiltration significantly contributes to the onset and progression of IS [5, 25]. Therefore, identifying specific diagnostic markers and analyzing the patterns of immune cell infiltration are of considerable significance for improving the prognosis of patients with IS. bioinformatics has provided a powerful strategy for screening molecular markers owing to the rapid developments in science and technology. In this study, we identified the diagnostic biomarkers of IS and investigated their immune infiltration patterns.

Ubiquitination plays an important role in IS [8, 26]. This study screened and identified 16 hub genes (AIM2, ZNF404, NANP, DHFR2, TRAPPC10, BCLAF3, CHAC2, SERPINB8, ZNF57, KHDC4, ZNF322, CDKN1A, TRMT1L, KBTBD6, DNAAF2, and PRKAR2B) from 33 ubiquitination-related genes having abnormal expression patterns. AIM2 is a cytoplasmic sensor that recognizes double stranded DNA [27]. AIM2 inflammasome is a protein platform in cells that cleaves precysteine protease-1 and transforms IL-18 and IL-1β into mature form to initiate innate immune response [27, 28]. An increasing number of studies have shown that the AIM2 inflammasome plays an important and decisive role in cardiovascular diseases, such as aortic aneurysm, coronary atherosclerosis, myocardial infarction, heart failure, ischemia/reperfusion injury and IS [27, 29–32]. ZNF404, ZNF57, and ZNF322 are transcriptional regulatory factors that are present in the nucleus and belong to the zinc-finger protein family. Zinc-containing transcription factors are the largest family of transcription regulatory factors in mammals and play important roles in processes, such as cell differentiation, proliferation, apoptosis, and tumor transformation [33]. ZNF404 may be involved in the activation of pathways related to vitamin C regulation of stem cell differentiation and proliferation [34]. ZNF322 is a novel human C (2) H (2) Kruppel-like zinc finger protein that exhibits the ability to modulate the transcriptional activation of the MAPK signaling pathway, a crucial mechanism implicated in the pathogenesis of IS [35]. Similarly, the overexpression of NANP, a transcription regulatory factor, promotes cell proliferation [36]. DHFR2 exists in the inner mitochondria membrane; it possesses reductase activity and is considered an optimized enzyme supporting vascular normalization during the critical window of embryonic development [37]. These findings indicate that the ubiquitination-related genes are involved in the occurrence and development of IS and may serve as potential early diagnostic markers of IS.

According to the ROC curve, the AUC value of AIM2 was 0.832, indicating that AIM2 had a strong ability to distinguish between the IS and control groups. AIM2 is a key driver of aseptic inflammatory responses in the brain and plays a role in innate immunity, cell death, and morphological changes in the neurons [38, 39]. In addition to traumatic brain injury and central nervous system infections, AIM2 inflammasomes have been reported to be associated with ischemic brain injury [40–42]. Habib et al. reported that the upregulation of AIM2 inflammasome expression after ischemic stroke can lead to brain injury and cognitive impairment in mice [43]. However, it is necessary to expand the sample size in future studies to further verify the effectiveness of AIM2 as a biomarker for IS.

GSEA provides valuable information on large-scale genes with minimal changes. By conducting GSEA on the gene profile of the dataset, we obtained numerous highly enriched gene sets from the IS group. The GO and KEGG annotation results indicated that IS mainly enriched the immune inflammation and apoptosis pathways, such as the Toll-like receptor signaling pathway, chemotherapy signaling pathway, T cell receptor signaling pathway, NOD-like receptor signaling pathway, apoptosis, and MAPK signaling pathway. Studies have revealed that the MAPK pathway is involved in regulating of various pathological and physiological processes in IS, including inflammation and apoptosis [44]. The MAPK pathway is activated In the early stages of cerebral ischemia [45]. In animal models of IS, the transcription of many pro-inflammatory molecules is mediated by p38, and these inflammatory factors can further activate p38, indicating the important role of p38 in mediating inflammation in IS [46]. The JNK signaling pathway plays a crucial role in regulating cell apoptosis after cerebral ischemia-reperfusion [47]. JNK activation exacerbates cellular inflammation and death in patients with stroke, leading to ischemic brain injury [48]. After 2 h of cerebral ischemia, the ERK pathway upregulates MMP expression, leading to the disruption of the blood-brain barrier. Furthermore, it simultaneously participates in the regulation of pro-inflammatory factors, exacerbating the inflammatory response [46].

We performed a comprehensive evaluation of immune cell infiltration using ssGSEA to further explore the role of immune cell infiltration in IS. Our findings revealed that the increased infiltration of type 1 T helper cell, type 17 T helper cell, eosinophil, and mast cell, as well as the decreased infiltration of memory CD4 T cell, activated B cell, and memory B cell, may be related to IS. Analysis of the correlation between hub genes and immune cells revealed that the expression levels of DHFR2 and DNAAF2 were significantly negatively correlated with eosinophil infiltration. Therefore, we speculated that the decreased expression of DHFR2 and DNAAF2 increased eosinophil infiltration, which is involved in IS pathophysiology. Eosinophils can secrete various chemokines and vascular endothelial growth factors, inducing the activation of M2 phenotype microglia, which have neuroprotective properties by promoting the resolution of inflammation [49–51]. Additionally, vascular endothelial growth factor may exert neuroprotective effects by regulating angiogenesis [49]. Eosinophils counts are independently associated with stroke severity and functional outcomes in patients with acute ischemic stroke [52]. Further studies are required to elucidate the complex interactions between these genes and immune cells.

This study successfully identified genes and signaling pathways significantly associated with ischemic stroke through the integrated application of various bioinformatics methods, offering new insights into the molecular mechanisms of the disease. However, there are some limitations. Firstly, the relatively small sample size may affect the reliability and generalizability of the results, as small sample sizes in complex diseases like IS can lead to biased findings. Secondly, the lack of clinical validation limits the findings to a theoretical level, with no immediate application in patient diagnosis and treatment. Future research should closely collaborate with clinical studies, using trials or cohort studies to validate the clinical relevance of these findings. Finally, the study did not incorporate wet-lab data, which may result in incomplete or inaccurate insights into certain mechanisms. Future research should integrate wet-lab data to confirm the accuracy of the bioinformatic analysis and uncover deeper molecular mechanisms. Addressing these limitations in future studies will enhance our understanding of the molecular mechanisms of IS and support the development of more effective diagnostic and therapeutic strategies.

## 5 Conclusions

This study identified DEGs, WGCNA modules, hub genes, enriched pathways, and infiltrating immune cells that may be closely associated with the pathogenesis of IS. These findings provide novel insights into the pathogenesis of IS and can be used to advance its treatment in the future.

## Supporting information

S1 Fig ROC curves of the hub genes. ROC curves of (A) TRMT1L. (B) KBTBD6. (C) DNAAF2. (D) PRKAR2B.
(TIF)

S1 Raw data (ZIP)
