# Supplementary figures and images for: Identifying immune cell infiltration and effective diagnostic biomarkers for ischemic stroke using bioinformatics analysis
Source: PLoS One. 2024 Dec 5;19(12):e0310108. doi: 10.1371/journal.pone.0310108 (PMC11620413; doi:10.1371/journal.pone.0310108)

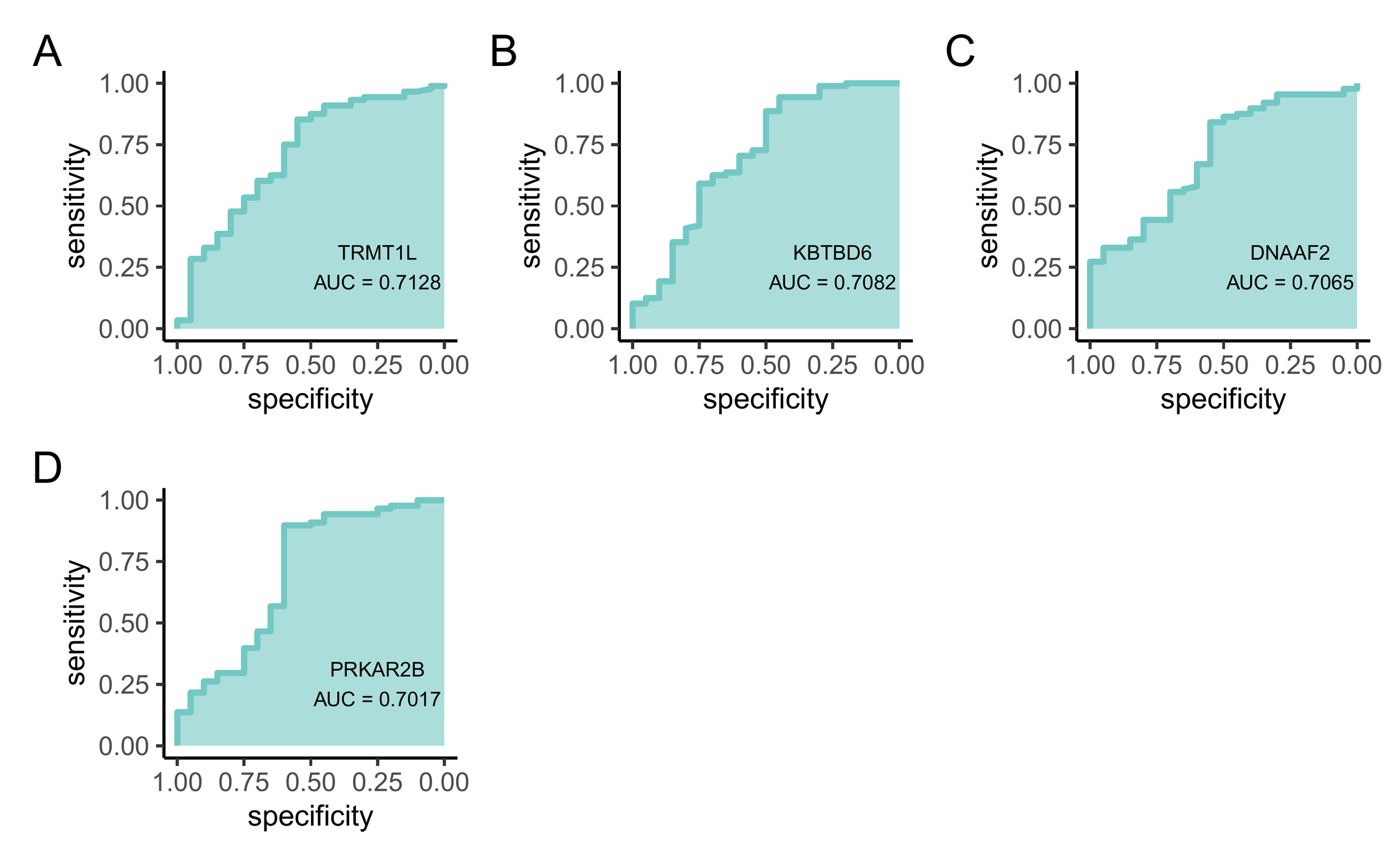

Supplement: S1 Fig — ROC curves of (A) TRMT1L. (B) KBTBD6. (C) DNAAF2. (D) PRKAR2B. (TIF) [file pone.0310108.s001.tif]
